# Investigating the effect of quadruple therapy with *Saccharomyces boulardii* or *Lactobacillus reuteri* strain (DSMZ 17648) supplements on eradication of *Helicobacter pylori* and treatments adverse effects: a double-blind placebo-controlled randomized clinical trial

**DOI:** 10.1186/s12876-022-02187-z

**Published:** 2022-03-07

**Authors:** Nooshin Naghibzadeh, Fatemeh Salmani, Samira Nomiri, Tahmine Tavakoli

**Affiliations:** 1 Gastroenterology Department, Faculty of Medicine, Birjand University of Medical Science, Birjand, Iran; 2 Epidemiology and Biostatistics Department, Social Determinants of Health Research Center, Faculty of Health, Birjand University of Medical Sciences, Birjand, Iran; 3 Clinical Biochemistry Department, Faculty of Medicine, Birjand University of Medical Sciences, Birjand, Iran

**Keywords:** Adverse effects, Eradication rate, *Helicobacter pylori*, *Lactobacillus reuteri*, *Saccharomyces boulardii*, Sequential treatment

## Abstract

**Background:**

The goal of this study was to investigate the effects of treatment with *Saccharomyces boulardii* and *Lactobacillus reuteri* on the eradication of *Helicobacter pylori* and Adverse effects (AEs) of the treatment.

**Results:**

This study was a double-blind, randomized, placebo-controlled trial. And, eradication of *H. pylori* was reported comparing quadruple therapy include of PPI (proton pomp inhibitor), bismuth subcitrate, clarithromycin, and amoxicillin versus quadruple therapy supplemented with *S. boulardii* and *L. reuteri* DSMZ 17648. For this aim, a total of 156 patients were included in the current study; and patients positive for *H. pylori* infection (n = 156) were randomly assigned to 3 groups: 52 patients (Group P) received conventional quadruple therapy plus *L. reuteri*, 52 patients (Group S) received conventional quadruple therapy plus *S. boulardii* daily, for 2 weeks, and 52 patients were in the control group (Group C). At the end of the treatment period, all the subjects continued to take proton pump inhibitor (PPI) alone for 14 days, and then, no medication was given for 2 weeks again. During follow-up, gastrointestinal symptoms were assessed using an evaluation scale (Glasgow dyspepsia questionnaire [GDQ]), and AEs were assessed at 7, 14, 21, and 28 days. As a result, all patients completed the treatment protocol in all groups by the end of the study. Additionally, eradication therapy was effective for 94.2% of subjects in Group S, 92.3% of subjects in Group P, and 86.5% of subjects in the control group, with no differences between treatment arms. In Group S, the chance of developing symptoms of nausea (OR = 2.74), diarrhea (OR = 3.01), headache (OR = 10.51), abdominal pain (OR = 3.21), and anxiety (OR = 3.58) was significantly lower than in the control group (*p* < 0.05).

**Conclusion:**

*S. boulardii* could significantly reduce some AEs of *H. pylori* eradication therapy, but effectiveness of *Lactobacillus reuteri* on these cases was not significant. It is recommended to conduct the future research with larger sample size in order to investigate the effect.

*Trial registration*: IRCT20200106046021N1, this trial was registered on Jan 14, 2020.

**Supplementary Information:**

The online version contains supplementary material available at 10.1186/s12876-022-02187-z.

## Introduction

*Helicobacter pylori* is a gram-negative infective bacterium affecting more than half of the world's population [1]. *H. pylori* infection contributes to etiology of a variety of diseases, such as gastric ulcer disease, dyspepsia, lymphoma, and gastric cancer [2–4]. Treatment of *H. pylori* infection is still being debated around the world, owing to emergence of multidrug-resistant strains of *H. pylori*. At the present, the most effective treatment options for *H. pylori* infection are complete pathogen eradication using multiple combinations of proton pump inhibitors (PPI) and two or three antibiotics [5–7]. This complicated approach carries a high risk of adverse effects (AEs), antibiotic resistance, and incompatibility [8, 9]. Reduced *H. pylori* load in the stomach by selective bacterial-bacterial surface interaction indicates a novel treatment approach to mitigating the danger posed by this human infection. Bacterial accumulation has previously been discussed in terms of infection elimination via specific binding to pathogens and formation of co-aggregates [10–12]. Many observations support the use of probiotics with bioactive components in people infected with *H. pylori*, recommending the use of multiplex probiotic microorganisms in control and treatment of infections caused by *H. pylori* [13, 14]. It has been proposed that Lactobacillus supplementation may be effective in accelerating removal of *H. pylori* in first-time patients, as well as having a positive effect on some AEs associated with *H. pylori* treatment [15]. To date, *saccharomyces boulardii* and several *Lactobacillus reuteri* strains have been used alone or in combination with various *H. pylori* treatment regimens in clinical studies [16–29]. Previous studies have explored the effects of *Saccharomyces boulardii* and *Lactobacillus Reuteri* on the eradication of *H. pylori* and the elimination of AEs associated with the four-drug treatment separately or in comparison. However, very few studies have been done to compare the effects of *Lactobacillus reuteri* (DSMZ 17648) and *Saccharomyces boulardii* on this topic.

Therefore, purpose of this study was investigating whether adding *saccharomyces boulardii* (DAILY EAST®) probiotic or a supplement of DSMZ 17648 *Lactobacillus reuteri* (PYLOSHOT®) to the standard quadruple treatment for *H. pylori* infection after 14 days increases eradication rate and reduces the AEs. Due to lack of sufficient studies in this field, there is a need for a clinical trial and it is hoped that the results of this study will be useful in improving treatment of *H. pylori*.

## Method and materials

A total of 156 patients were included in the current study. A total of 156 patients were included in the current study. The sample size was calculated based on Zojaji et al. [27] by using a sample size formula for comparison proportion. In this study, the percentage of improvement in group A (amoxicillin 1 g twice daily, clarithromycin 500 mg twice daily, omeprazole 20 mg twice daily, and probiotic Saccharomyces bulardi 250 mg twice daily) was 0.875, and in group B (amoxicillin 1 g twice daily, clarithromycin 500 mg twice daily, omeprazole 20 mg twice daily) was 0.812.

$$\begin{aligned} n & = \frac{{\left( {z_{{1 - \frac{\alpha }{2}}} \sqrt {\left( {2\overline{p}\left( {1 - \overline{p}} \right)} \right)} + z_{\beta } \sqrt {p_{1} \left( {1 - p_{1} } \right) + p_{2} \left( {1 - p_{2} } \right)} } \right)^{2} }}{{\left( {p_{1} - p_{2} } \right)^{2} }} \\ & = \frac{{\left( {1.96\sqrt {2*0.8435*(1 - 0.8435)} + 0.84\sqrt {0.875(1 - 0.875) + 0.812(1 - 0.812)} } \right)^{2} }}{{\left( {0.2} \right)^{2} }} \approx 52 \\ \end{aligned}$$

At the end of the treatment period, all the subjects continued to take proton pump inhibitor (PPI) alone for 14 days, and then no medication was given for 2 weeks again. During follow-up, gastrointestinal symptoms were assessed using an evaluation scale (Glasgow dyspepsia questionnaire (GDQ)), and Adverse effects were assessed at 7, 14, 21, and 28 days.

### Study design

**Fig. 1:**
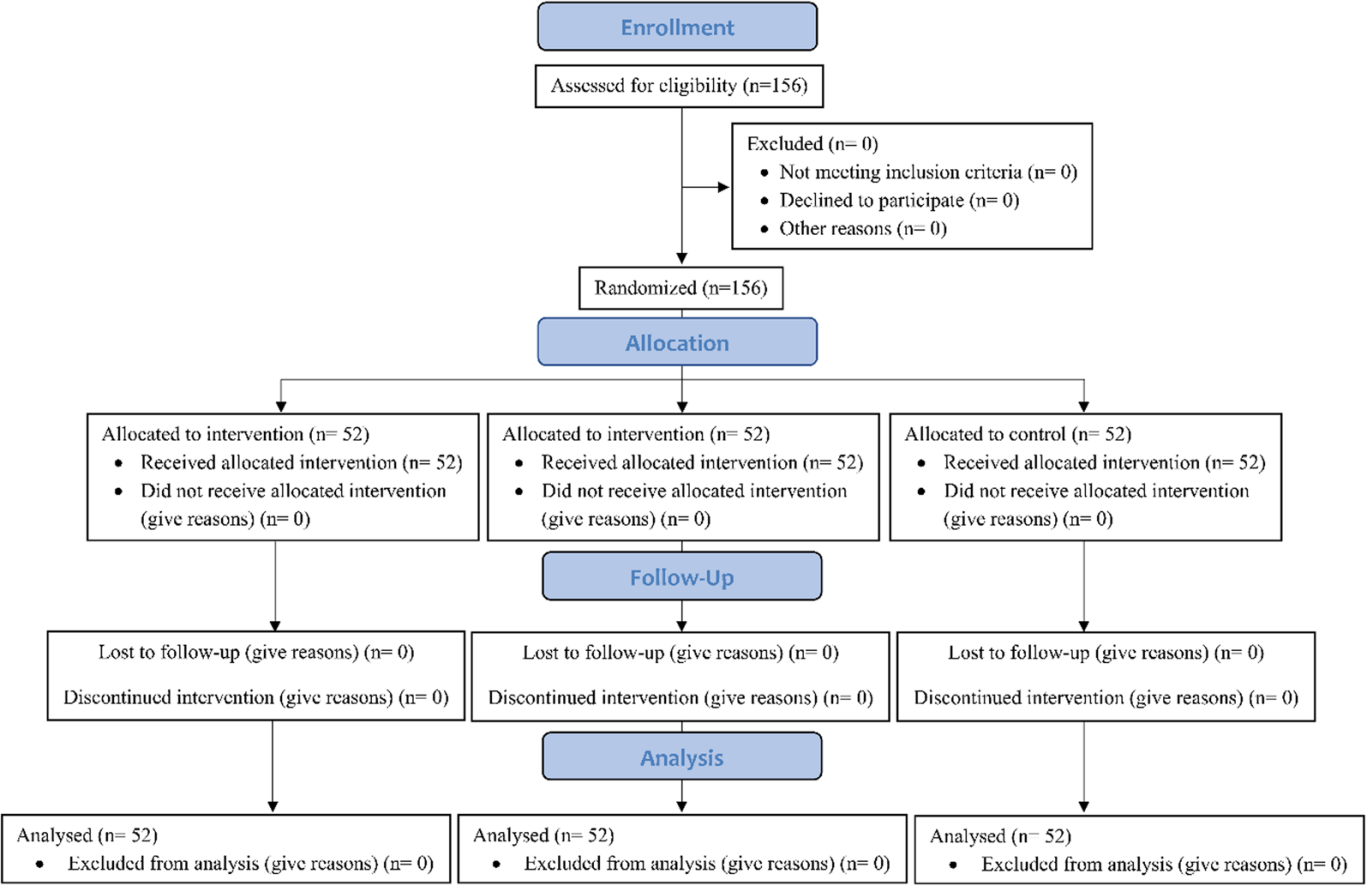
CONSORT diagram of the study

This study was designed according to the Consolidated Standards of Reporting Trials (CONSORT) guideline [30]. The work flow for the overall procedure is shown in Fig. 1. Patients who were referred to the Gastroenterology Clinic of the Valiasr Hospital and met all the inclusion criteria were included in the study. Then, patients were randomly assigned to treatment groups by tossing a coin.

#### Inclusion criteria

Symptomatic *H. pylori*-positive patients of both sexes aged between 16 and 74 years old whose infection was confirmed by endoscopy and pathology were included in the study. Endoscopy and histopathological analysis on Antrum and body were performed by a pathologist blinded to the treatment arm and the time points of tissue sampling to determine the presence of *H. pylori*. Giemsa stain was used to stain all biopsies.

#### Exclusion criteria

Patients with less than 15 years of age, pregnant or lactating women, patients with hepatic, cardio-respiratory, renal, and neoplastic diseases, those receiving antibiotics, PPIs, bismuth salts, or probiotics within the previous 4 weeks, patients with a history of gastric surgery and sensitivity to any of the drugs used in this study, those receiving any medication interfering with the action of the lactobacilli, gastrectomy or gastric bypass, patients with autoimmune disease, organ transplantation, weight changes > 3 kg over the last 3 months, those with the history of eradication of *H. pylori* infection, lactose intolerance, and those participating in other clinical trials at the same time were excluded from the study.

### Ethical considerations

This study was approved by the Research Council and Ethics Committee of the Birjand University of Medical Sciences with the ethics code of IR.BUMS.REC.1398.305. It was also registered on the website of the Iranian registry of clinical trials (IRCT) with the following Number: IRCT20200106046021N1 on 14/01/2020. An informed written consent was obtained from all the patients. No costs were imposed on patients. The drugs used in the study, as well as the dose administered to patients, had no AEs or toxicity. The final report and analysis were performed without names of the participants in the study. The modified Glasgow dyspepsia questionnaire (GDQ) was used to record dyspeptic symptoms before starting therapy and at the end of the follow-up period.

### Treatment protocol

The interventions were based on the regimen containing bismuth and clarithromycin, and amoxicillin for *H. pylori*, which included 1 g of Amoxicillin twice daily, 500 mg of Clarithromycin twice daily, 40 mg of PPI (esomeprazole (Nexium®), lansoprazole (Takepron®. OD), pantoprazole (Pantoloc®), and rabeprazole(pariet)) twice daily, and 120 mg of bismuth subcitrate (two tablets, twice daily). Patients in Group S were given 1 capsule of DAILYEAST® (*saccharomyces boulardii* supplement 250 mg, ZistTakhmir, Tehran, Iran) b.i.d in combination with anti *H. pylori* quadruple therapy. Patients in Group P were given 2 capsules of PHYLOSHOT® (100 mg of non-viable *Lactobacillus reuteri* DSMZ 17648, Lactobacillus acidophilus, Lactobacillus casei, and Bifidobacterium lactis > 10^9^ CFU, ZistTakhmir, Tehran, Iran) b.i.d in combination with anti *H. pylori* quadruple therapy. Patients in the control group were also given a placebo capsule b.i.d along with anti *H. pylori* quadruple therapy. Duration of treatment was equal to 14 days.

**Fig. 2:**
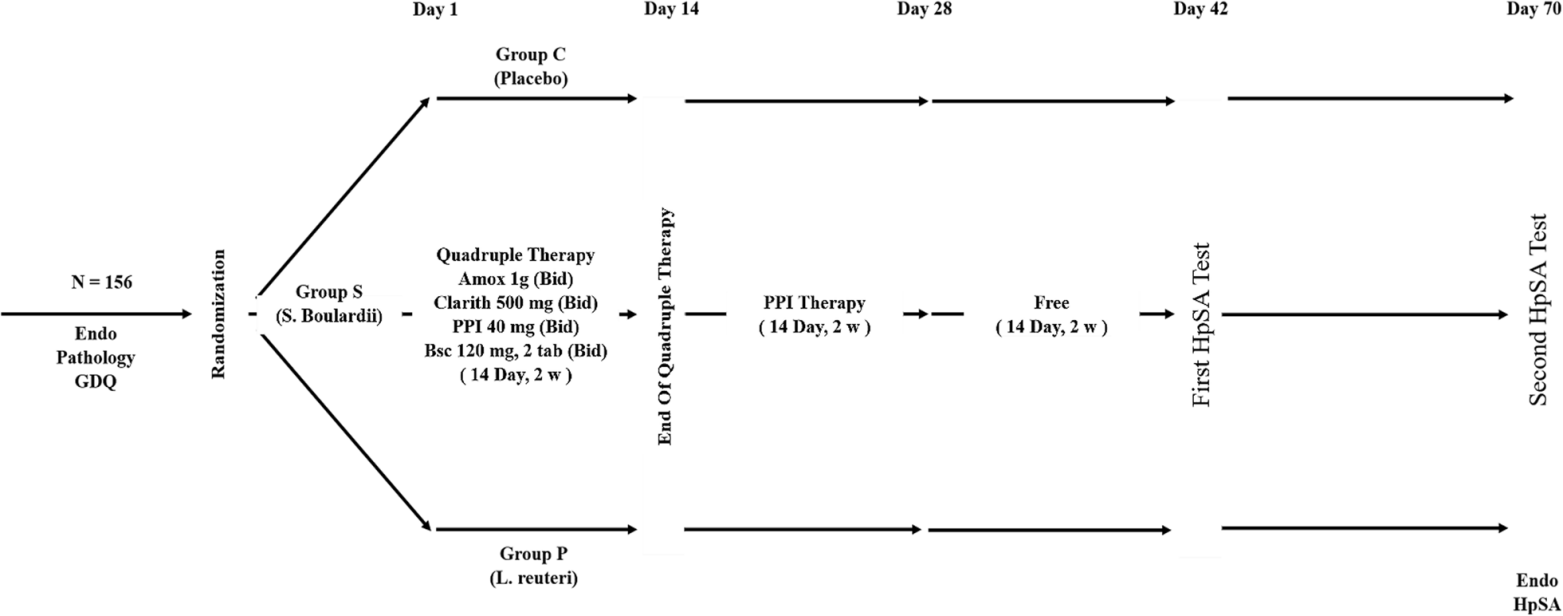
Flow chart of the study. A total of 156 patients met the inclusion criteria. HpSA: *H. pylori* stool antigen, Endo: endoscopic, GDQ: Glasgow dyspepsia questionnaire, PPI: proton pomp inhibitor, Amox: amoxicillin, Clarith: clarithromycin, b.i.d: twice daily, BSC: bismuth subcitrate

At the end of the treatment period, all the subjects continued to take PPI alone for 14 days. Then, the patients were given no medication for 2 weeks before having their first fecal antigen test (Novin Research®), at the end of the second week. Then, after another 4 weeks, they had a second stool antigen test (Fig. 2).

### Evaluation of treatments adverse effects and tolerability

Using a previously reported questionnaire, the Adverse effects (AEs) profile and tolerability were assessed during the follow-up period. The term "Adverse effects" in this study refers to the symptoms and complaints of patients caused by the use of antibiotic medicines during treatment and their persistence until two weeks after stopping antibiotics. Subjects were carefully instructed and trained on filling out questionnaires in order to achieve the highest level of compliance in registering any potential treatment-related AEs. Subjects were asked to report any side effects during and after therapy, such as bitter taste, nausea, vomiting, epigastric discomfort, abdominal pain, and diarrhea.

### Statistical analysis

After entering the SPSS 24 software, the data were described using central and dispersion indices for quantitative variables and frequency and agreement tables for qualitative variables. Homogeneity of groups was assessed using analysis of variance (ANOVA) and Chi-Square tests. Marginal logistic model with generalized estimating equation (GEE) approach was used for modeling the changes in complications during the study. In the tests, significance level was set at 0.05.

## Results

### Patients

A total of 156 subjects with confirmed *H. pylori* infection completed the treatment protocol in all groups by the end of the study. The study included 60.9 percent female participants and 39.1% male participants, with a mean age of 47.76 ± 13.92 years old (age range of 16–74 years old). They were randomly assigned into three study arms: 52 patients were assigned into the *L. Reuteri* group (Group P), 52 patients were assigned into the *S. boulardii* group (Group S), and 52 patients were assigned into the placebo group (Group C). The majority of our patients (93.6%) were from rural areas, housewife (46.8%), and non-smokers (76.9%). Dyspepsia was the most common reason for undergoing an endoscopy procedure (64.7%). At the time of the first endoscopy, the majority of patients had antral gastritis. Antral and corpus predominant gastritis were found in 88.5and 27.6% of patients, respectively. The three treatment groups were similar in terms of their demographic, clinical, and endoscopic characteristics at baseline (Table 1) (*p* > 0.05).

**Table 1:** Characteristics of the all groups

	Group C	Group S	Group P	*p *value
*Age (years)*				
Mean ± SD	46.57 ± 14.20	49.32 ± 13.42	47.38 ± 14.25	0.58
*Sex*				
Male	34.6% (18)	48.1% (25)	34.6% (18)	0.26
Female	65.4% (34)	51.9% (27)	65.4% (34)	
*Smoking*				
Yes	17.3% (9)	32.7% (17)	19.2% (10)	0.12
No	82.7% (43)	67.3% (35)	80.8% (42)	
*Endoscopic reasons*				
Dyspepsia	59.6% (31)	67.3% (35)	67.3% (35)	0.63
Heart Burn	25% (13)	25% (13)	21.2% (11)	0.86
*Endoscopic pattern of gastritis*				
Antral gastritis	92.3% (48)	82.7% (43)	90.4% (47)	0.26
Corpus gastritis	23.1% (12)	21.2% (11)	38.5% (20)	0.09
Pan-gastritis	3.8% (2)	11.5% (6)	3.8% (2)	0.22
Duodenal ulcer	3.8% (2)	0.0% (0)	1.9% (1)	0.77
Gastric ulcer	0.0% (0)	1.9% (1)	5.8% (3)	0.32
Esophagitis	0.0% (0)	0.0% (0)	0.0% (0)	–
Normal	1.9% (1)	1.9% (1)	0.0% (0)	1.00

### Eradication rate

**Fig. 3:**
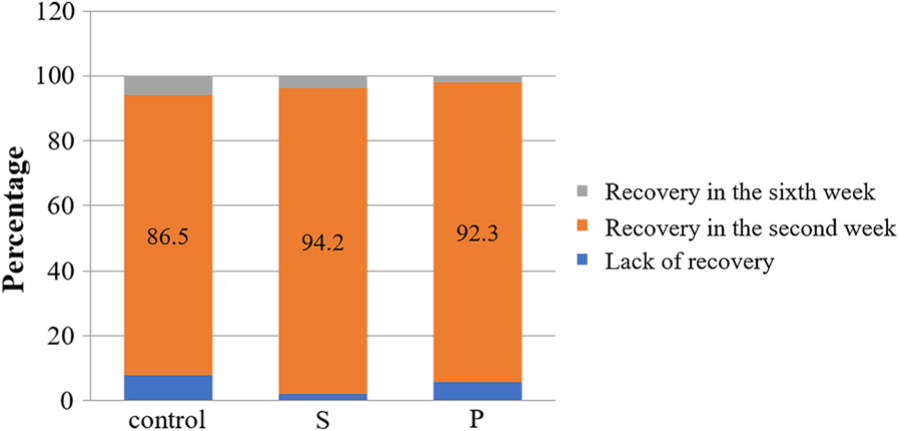
Rate of eradication of *H. pylori* infection in the studied groups

The highest rate of *H. pylori* eradication occurred in all groups in the second week after treatment. Totally, 86.5, 94.2, and 92.3% of eradication was observed in the control, S, and P groups, respectively. In general, eradication rate in the studied groups was not statistically significant in the second and sixth weeks, (with *P* = 0.46 in the second week and *P* = 0.53 in the sixth week, respectively) (Fig. 3).

### Patients compliance and adverse effects

**Fig. 4:**
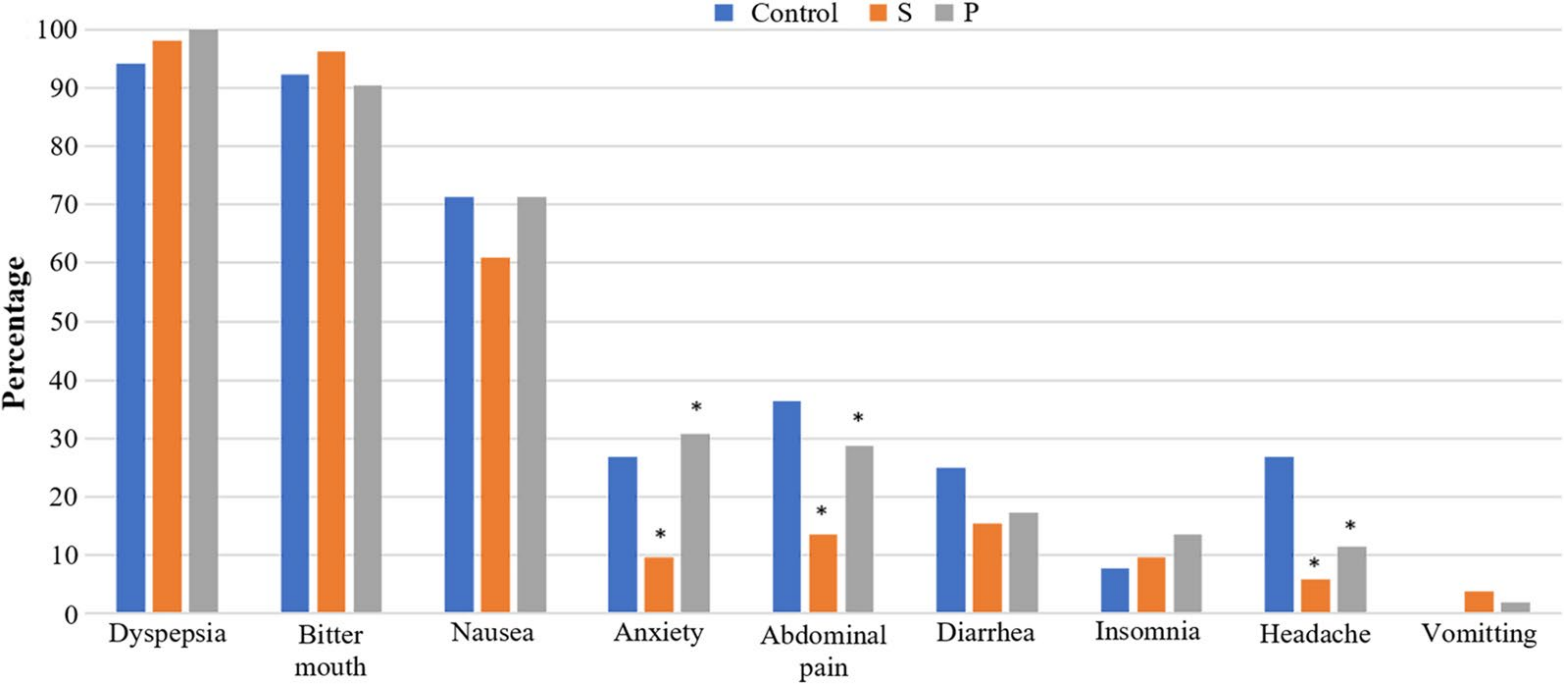
Distribution frequency of AEs observed in the study groups

Headache, abdominal pain, and anxiety were significantly reduced in the Group S (who received *saccharomyces boulardii* probiotic-DAILYEAST®). But in Group P, headache and abdominal pain were significantly reduced and anxiety was significantly increased (*p* < 0.05). However, none of the study groups experienced significant changes in vomiting, insomnia, bitter taste in the mouth, or epigastric discomfort (*p* > 0.05) (Table 2). According to Table 2, after adjusting the effect of time, in the Group S, chance of developing symptoms of headache (OR = 10.51), abdominal pain (OR = 3.21), and anxiety (OR = 3.58) was significantly lower than control group (*p* < 0.05). Also, except for headache (OR = 3.75), the Group P did not differ significantly from the control group in incidence of complications (*p* > 0.05) (Fig. 4). For more details, see Additional file 1.

**Table 2:** Generalized estimating equations (GEE) logistic model of the association between the treatment group and complications

Outcome	Variables		Coefficient	SE	*P* value	OR
Nausea	Treatment group	S	0.90	0.36	0.01	2.47
	Base line: control group	P	0.41	0.36	0.26	1.52
	Time		1.51	0.14	< 0.001	4.55
Diarrhea	Treatment group	S	1.10	0.49	0.02	3.01
	Base line: control group	P	0.63	0.51	0.21	1.89
	Time		1.74	0.16	< 0.001	5.74
Headache	Treatment group	S	2.35	0.65	< 0.001	10.51
	Base line: control group	P	1.32	0.55	0.01	3.75
	Time		1.08	0.26	< 0.001	2.95
Abdominal pain	Treatment group	S	1.16	0.54	0.03	3.21
	Base line: control group	P	0.39	0.43	0.36	1.48
	Time		1.14	0.17	< 0.001	3.14
Anxiety	Treatment group	S	1.27	0.56	0.02	3.58
	Base line: control group	P	− 0.38	0.46	0.40	0.68
	Time		0.61	0.12	< 0.001	1.86

## Discussion

Treatment of *H. pylori* infection is becoming increasingly important, particularly in developing countries. Despite availability of various therapeutic regimens, treatment failure has remained a growing problem in daily clinical practices. Several factors could contribute to failure of eradication, but the most important factors are antibiotic resistance and clinical efficacy [31]. According to findings of the current study, there was no statistically significant difference between the study results at the second and sixth weeks after treatment. These findings were in agreement with the Zojaji et al. study that evaluated the efficacy and safety of adding *S. boulardii* to standard triple therapy in 160 adult patients with biopsy confirmed *H. pylori* infection. They randomized patients into two treatment regimens: patients in group A (n = 80) were given amoxicillin, clarithromycin, omeprazole, and a probiotic of *Saccharomyces boulardii* for 14 days. Moreover, patients in group B (n = 80) were given amoxicillin, clarithromycin, and omeprazole for 14 days. After the second week, the success rate for *H. pylori* eradication in group A was higher at 75 (87.5%) than in group B at 65 (81.2%), but the difference between the two groups was not significant [27]. Furthermore, Cindoruk et al., in a study on assessing the effect of *S. boulardii* on eradication of *H. pylori* infection and reduction of AEs reported no significant difference in *H. pylori* eradication between the study groups (71% in the *S. boulardii*-treated group and 60% in the placebo group), which was consistent with our findings [32]. Also, Shavakhi et al., found that using a combination of probiotics containing Lactobacillus, Bifidobacterium, and *Streptococcus thermophilus* species along with a standard quadruple therapy had no beneficial effect in treating *H. pylori* infection. This could be due to the probiotic diet's low dose or high frequency of upper gastrointestinal AEs, which can reduce *H. pylori* eradication [33]. On the other hand, Poonyam et al., indicated that eradication of *H. pylori* was significantly increased compared to the control group when *Lactobacillus reuteri* was used in combination with a standard quadruple therapy [34]. Moreover, Yu et al. demonstrated that Lactobacillus can significantly eradicate *H. pylori* in a meta-analysis with a sample size of 724 patients to investigate the probiotic effect of Lactobacillus in combination with a triple eradication regimen [35]. In addition, Zhou et al., in a meta-analysis showed that *S. boulardii* can significantly increase eradication of *H. pylori* [36], all these studies were not in agreement with our findings. Among the reasons for this mismatch, one can mention a difference in the type of probiotic used in the studies in terms of the strains used or the large sample size and multi-center nature of these studies are reasons for the inconsistency between the findings.

With the widespread use of probiotics in clinical practice in recent years, the concept of "treating bacteria with probiotics" has been proposed as a new strategy to eradicate *H. pylori*. However, the mechanism underlying *L. reuteri* and S. Bouvardia’s eradication of *H. pylori* has not been fully elucidated. Several potential mechanisms have been proposed, including the following:

1. The volume of *S. boulardii* is 10 times larger than that of common bacterial strains of probiotics, which increases the surface area and can better adhere to pathogenic bacteria, affecting the colonization of *H. pylori* in the gastric mucosa [37]. *S. boulardii* comprises neuraminidase activity selective for alpha (2–3)‐linked sialic acid, and acts as a ligand binding to *H. pylori* adhesin, which in turn inhibits the adhesion of *H. Pylori* in the duodenum [38].
2. Increasing antimicrobial substances, such as short‐chain fatty acids, inhibiting the growth and proliferation of *H. pylori* [39].
3. *S. boulardii* may stimulate an increase in secretory IgA and immunoglobulin levels [40], strengthening the mucosal immune barrier. *H. pylori* infection can stimulate gastric epithelial cells to produce inflammatory mediators such as interleukin (IL) and tumor necrosis factor (TNF) [41, 42].
4. Reducing the incidence of adverse effects and, subsequently, compliance may improve, which may indirectly improve the *H. pylori* eradication rates.

In the case of *L. reuteri*, In vitro and in artificial gastric juice, *H. pylori* specifically co-aggregates with *H. pylori* without interfering with other commensal intestinal flora bacteria [43]. This specific binding may obscure *H. pylori* surface structures and impair Helicobacter motility. Pathogens that have aggregated are presumably no longer adhered to the gastric mucosa and are cleared from the stomach. Competition for specific binding proteins could be another mode of action [44].

According to findings of our study, distribution frequency of some AEs in the subjects receiving *S. boulardii* probiotic was significantly reduced after the intervention but, except for headache, no significant change was observed in the *Lactobacillus reuteri*-treated group. According to the findings, the most common AEs in the first week were vomiting, bitter taste in the mouth, epigastric discomfort, and insomnia, which were decreased after that time. However, no statistically significant difference was found between the studied groups at different times. Our findings were consistent with those of the studies by Pourmasoumi et al., and Lv et al., who showed that probiotic administration can reduce the AEs of anti *H. pylori* treatment [26, 28]. Also, Zojaji et al., discovered that using *S. boulardii* supplement significantly reduces AEs, such as nausea, diarrhea, epigastric discomfort, and bloating in the first and second weeks of treatment [27]. Furthermore, Cindoruk et al., in a study found that the AEs of epigastric discomfort and dyspepsia were significantly reduced in the group that received *S. boulardii* [32]. Moreover, Shavakhi et al., reported that incidence of diarrhea was significantly lower in the group that consumed combined probiotics containing Lactobacillus, Bifidobacterium, and Streptococcus thermophilus than the placebo group. On the other hand, incidence of abdominal pain was significantly higher in the probiotic group than the placebo group [33]. On the contrary, Frequency of nausea, vomiting, epigastric discomfort, and bitter taste was significantly reduced in the group that received oral supplement of *Lactobacillus reuteri* compared to the placebo group in the study by Poonyam et al. [34]. Also, Yu et al., in a meta-analysis found that Lactobacillus reduces treatment-related AEs [35]. Furthermore, Zhou et al., discovered that *S. boulardii* could significantly reduce treatments AEs, particularly diarrhea and constipation [36]. which were not in line with the results of our study.

## Limitations of the study

One of the study's limitations is that it does not investigate the severity of Adverse effects caused by antibiotics. Furthermore, the term "Adverse effects" in this study is defined differently than its usual definition and refers to patients' symptoms and complaints about the side effects of the antibiotics during treatment and their persistence until two weeks after stopping antibiotics. Furthermore, in our country, simply receiving the code of ethics is sufficient to begin research, and for this reason, we began collecting samples after the ethics committee's approval.

## Conclusion

In general, our findings revealed that the use of probiotic supplements containing *S. boulardii* could significantly reduce some of the AEs of *H. pylori* eradication therapy. But, the effectiveness of *Lactobacillus reuteri* (DSMZ 17648 strain) on these cases was not significant, and only the headache was remarkably reduced, which was in accordance with the previous evidence in the literature. Therefore, it is recommended to conduct future research with a larger sample size in order to investigate the effect of *S. boulardii* supplementation on eradicating *H. pylori* infection and reducing treatment AEs.

## Supplementary Information

**Additional file 1.** Supplementary figures: The frequency distribution of side effects over time in the study groups.

## Data Availability

The datasets used and analyzed during the current study are available from the corresponding author on reasonable request.
