# Supplementary material for: Investigating the effect of quadruple therapy with Saccharomyces boulardii or Lactobacillus reuteri strain (DSMZ 17648) supplements on eradication of Helicobacter pylori and treatments adverse effects: a double-blind placebo-controlled randomized clinical trial
Source: BMC Gastroenterol. 2022 Mar 7;22:107. doi: 10.1186/s12876-022-02187-z (PMC8903632; doi:10.1186/s12876-022-02187-z)
Supplement: Supplementary file 1 — Additional file 1. Supplementary figures: The frequency distribution of side effects over time in the study groups. [file 12876_2022_2187_MOESM1_ESM.docx]

# **Supplementary figures: The frequency distribution of side effects over time in the study groups**

Corresponding author:

**TahmineTavakoli,** Gastroenterology Department, Faculty of Medicine, Birjand University of Medical Science, Birjand, Iran. Tel: +98 56 3162 2035;

Email: tahminetavakoli95238@yahoo.com.


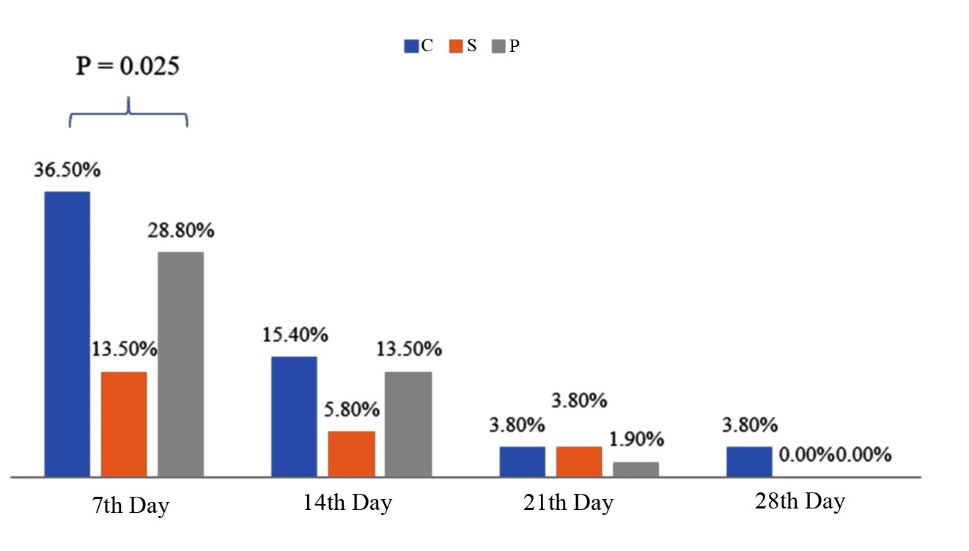


**Figure S1:** **Abdominal pain**. Frequency distribution of abdominal pain side effect over time in the study groups (C= control group, P= conventional quadruple therapy plus L. reuteri, S= conventional quadruple therapy plus S. boulardii daily, for 2 weeks). As Shown, there was a significant difference between groups in 7th Day (P<0.05).

**
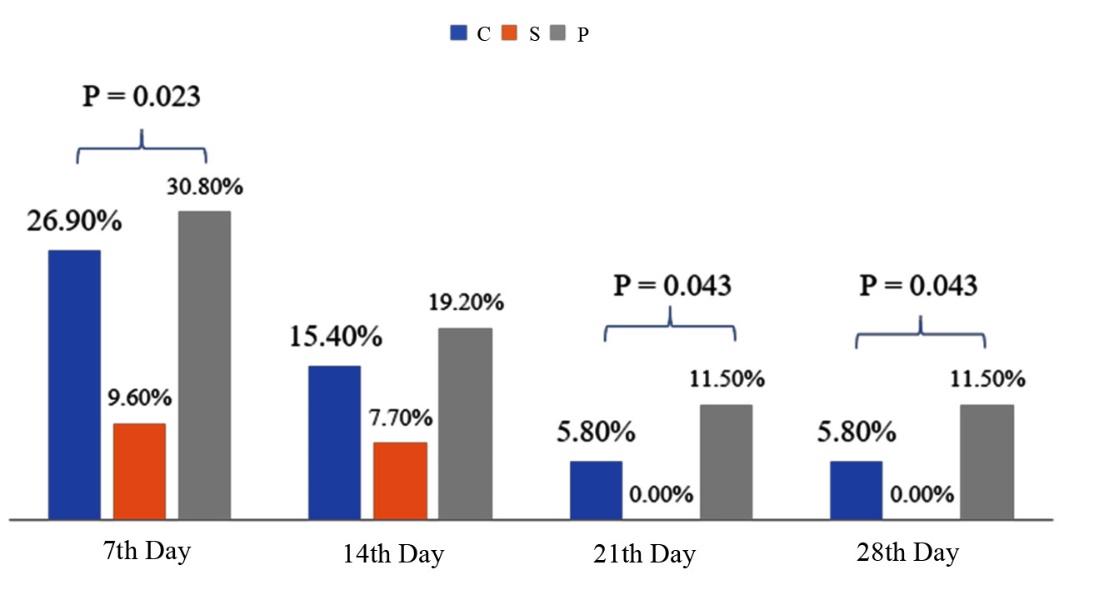
**

**Figure S2:** **Anxiety**. Frequency distribution of anxiety side effects over time in the study groups (C= control group, P= conventional quadruple therapy plus L. reuteri, S= conventional quadruple therapy plus S. boulardii daily, for 2 weeks). As Shown, there were significant difference between groups in 7th Day, 21th Day and 28th Day (P<0.05).

**
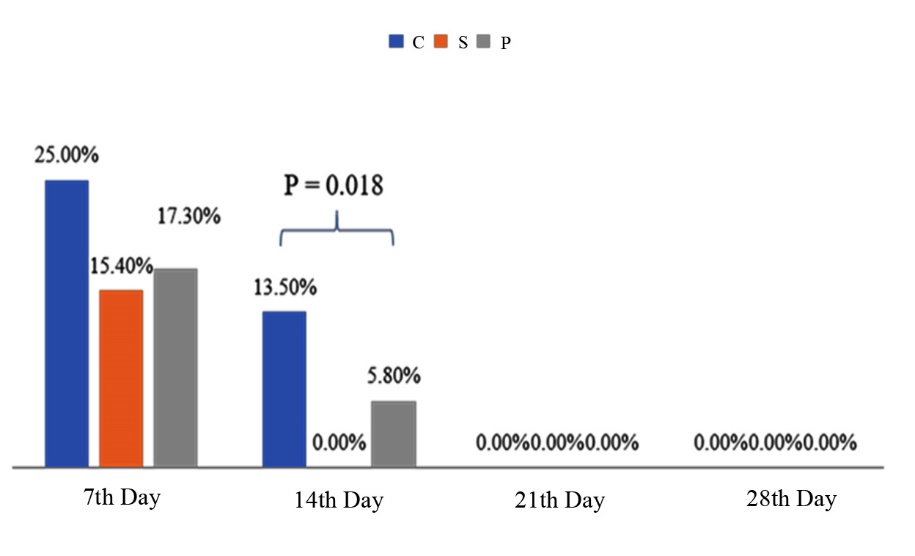
**

**Figure S3:** **Diarrhea**. Frequency distribution of diarrhea side effect over time in the study groups (C= control group, P= conventional quadruple therapy plus L. reuteri, S= conventional quadruple therapy plus S. boulardii daily, for 2 weeks). As Shown, there was a significant difference between groups in 14th Day (P<0.05).

**
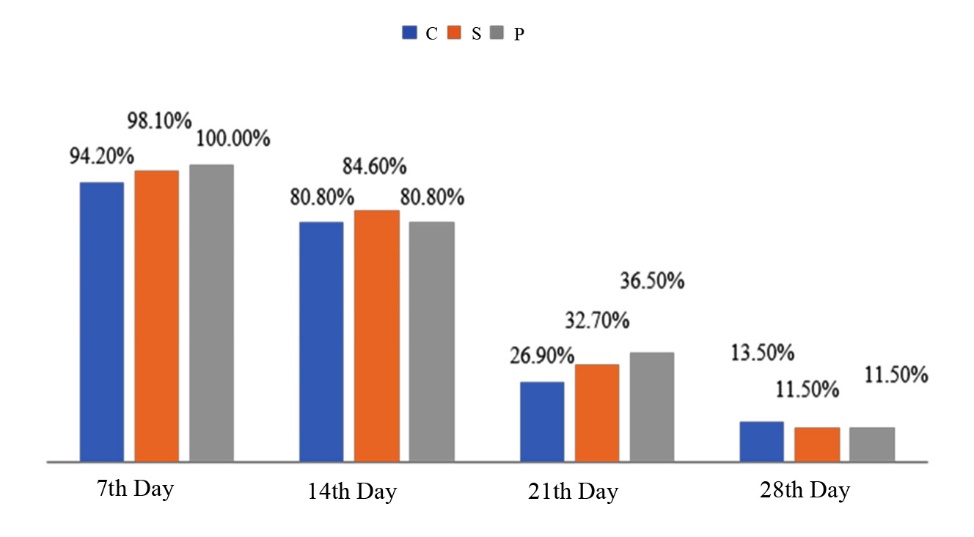
**

**Figure S4:** **Epigastric discomfort.** Frequency distribution of epigastric discomfort side effect over time in the study groups (C= control group, P= conventional quadruple therapy plus L. reuteri, S= conventional quadruple therapy plus S. boulardii daily, for 2 weeks). As Shown, there was no significant difference between groups.

**
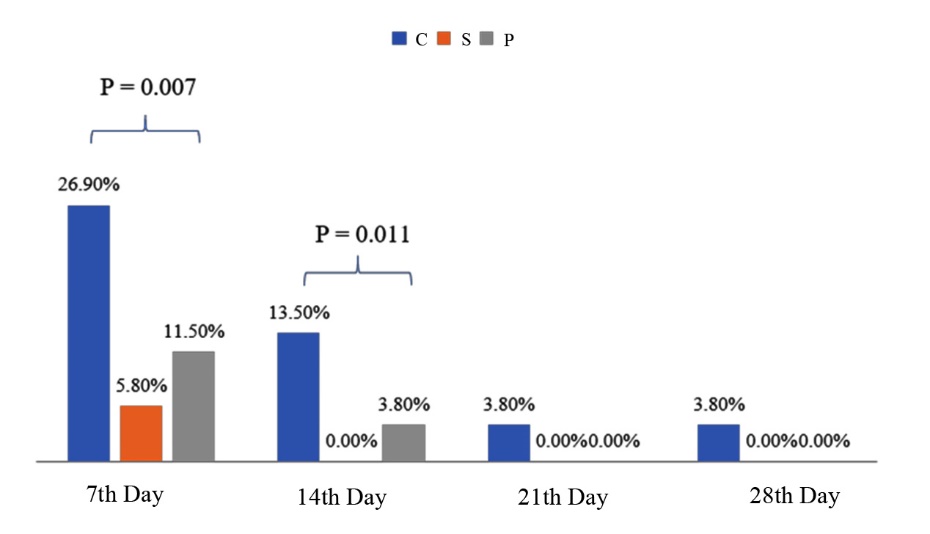
**

**Figure S5:** **Headache.** Frequency distribution of headache side effect over time in the study groups (C= control group, P= conventional quadruple therapy plus L. reuteri, S= conventional quadruple therapy plus S. boulardii daily, for 2 weeks). As Shown, there were significant difference between groups in 7th Day and 14th Day(P<0.05).

**
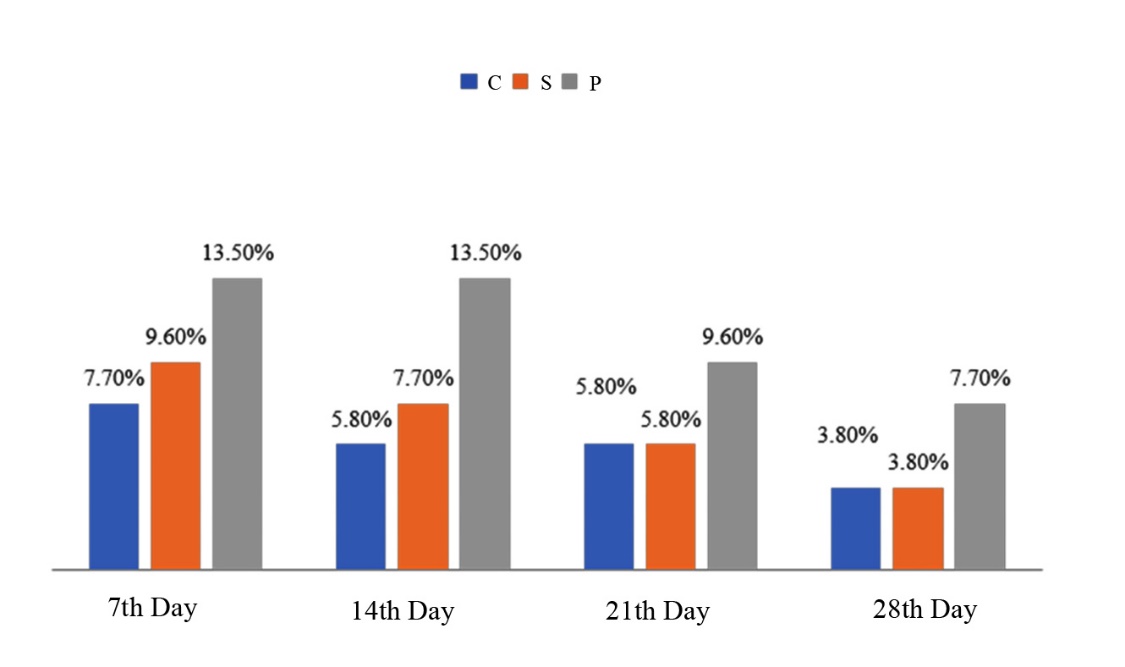
**

**Figure S6:** Insomnia. Frequency distribution of insomnia side effect over time in the study groups (C= control group, P= conventional quadruple therapy plus L. reuteri, S= conventional quadruple therapy plus S. boulardii daily, for 2 weeks). As Shown, there was no significant difference between groups.

**
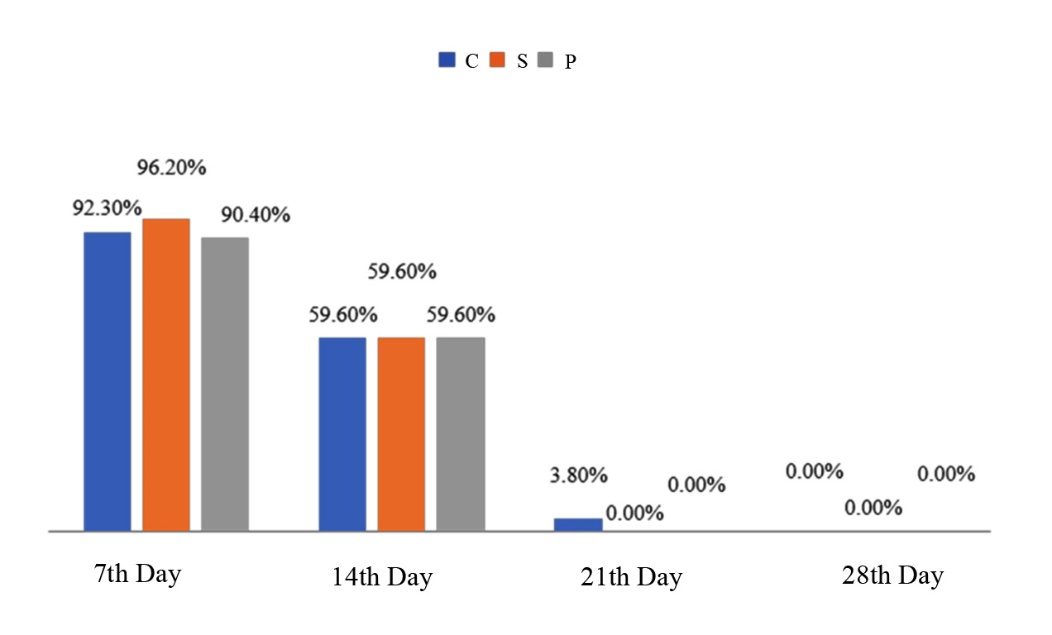
**

**Figure S7:** **Metallic taste sensation in the mouth**. Frequency distribution of metallic taste sensation in the mouth over time in the study groups (C= control group, P= conventional quadruple therapy plus L. reuteri, S= conventional quadruple therapy plus S. boulardii daily, for 2 weeks). As Shown, there was no significant difference between groups.

**
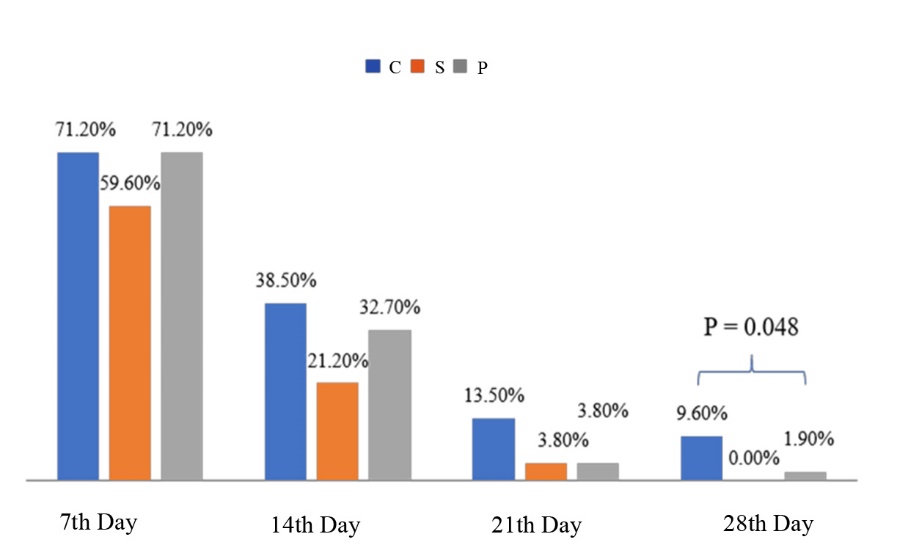
**

**Figure S8:** **Nausea**. Frequency distribution of nausea side effect over time in the study groups (C= control group, P= conventional quadruple therapy plus L. reuteri, S= conventional quadruple therapy plus S. boulardii daily, for 2 weeks). As Shown, there was a significant difference between groups in 28th Day (P<0.05).

**
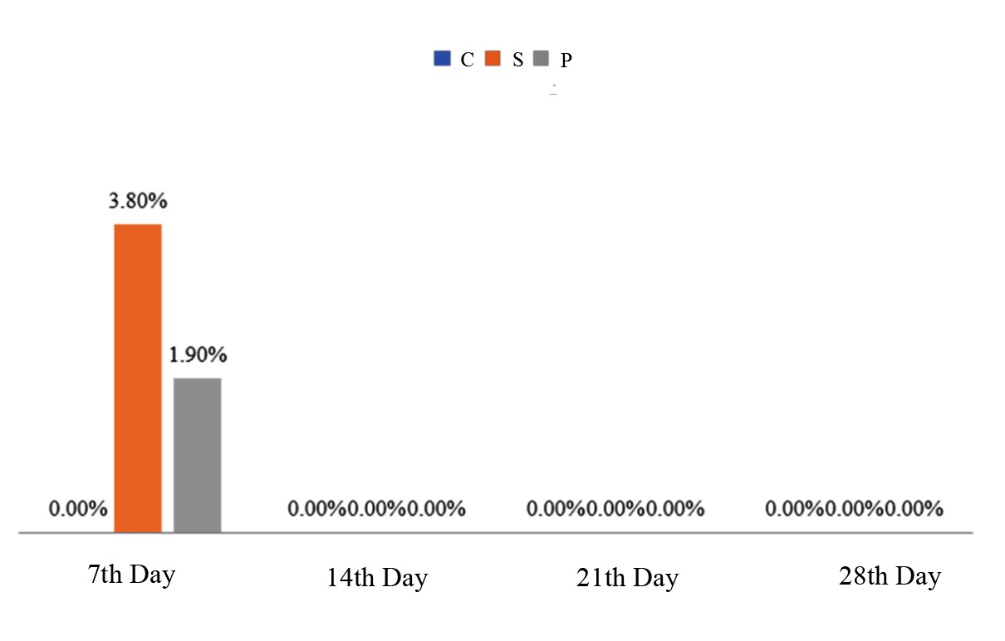
**

**Figure S9:** **Vomiting.** Frequency distribution of vomiting side effect over time in the study groups(C= control group, P= conventional quadruple therapy plus L. reuteri, S= conventional quadruple therapy plus S. boulardii daily, for 2 weeks). As Shown, there was no significant difference between groups.
